# Presence of brain metastasis differentially impacts long-term survival after first-line therapy in melanoma depending on BRAF mutation status

**DOI:** 10.3389/fimmu.2025.1536642

**Published:** 2025-02-14

**Authors:** Jan-Malte Placke, Luisa Sophie Rajcsanyi, Rudolf Herbst, Patrick Terheyden, Jochen Utikal, Claudia Pföhler, Alexander Kreuter, Peter Mohr, Ralf Gutzmer, Michael Weichenthal, Friedegund Meier, Carola Berking, Ulrike Leiter, Johanna Seier, Frederik Krefting, Alpaslan Tasdogan, Georg C. Lodde, Elisabeth Livingstone, Lisa Zimmer, Alexander Roesch, Klaus Griewank, Dirk Schadendorf, Selma Ugurel

**Affiliations:** ^1^ Department of Dermatology, University Hospital Essen, Essen, Germany; ^2^ German Consortium for Translational Cancer Research (DKTK), partner site Essen/Düsseldorf, Essen, Germany; ^3^ Section of Molecular Genetics in Mental Disorders, University Hospital Essen, Essen, Germany; ^4^ Center for Translational Neuro- and Behavioral Sciences, University Hospital Essen, Essen, Germany; ^5^ Institute for Sex- and Gender-Sensitive Medicine, University Hospital Essen, Essen, Germany; ^6^ Department of Dermatology, Helios Klinikum Erfurt, Erfurt, Germany; ^7^ Department of Dermatology, University of Lübeck, Lübeck, Germany; ^8^ Skin Cancer Unit, German Cancer Research Center (DKFZ), Heidelberg, Germany; ^9^ Department of Dermatology, Venereology and Allergology, University Medical Center Mannheim, Ruprecht-Karl University of Heidelberg, Mannheim, Germany; ^10^ DKFZ-Hector Cancer Institute at the University Medical Center Mannheim, Mannheim, Germany; ^11^ Department of Dermatology, Saarland University Medical School, Homburg/Saar, Germany; ^12^ Department of Dermatology, Venereology and Allergology, HELIOS St. Elisabeth Klinik Oberhausen, University Witten-Herdecke, Oberhausen, Germany; ^13^ Department of Dermatology, Elbe Kliniken Buxtehude, Buxtehude, Germany; ^14^ Department of Dermatology, Skin Cancer Center Minden, Minden, Germany; ^15^ Department of Dermatology, University Hospital of Schleswig-Holstein, Kiel, Germany; ^16^ Department of Dermatology, Faculty of Medicine and University Hospital Carl Gustav Carus, Technische Universität Dresden, Dresden, Germany; ^17^ Department of Dermatology, Uniklinikum Erlangen, CCC Erlangen – EMN, Friedrich-Alexander-Universität Erlangen-Nürnberg, Erlangen, Germany; ^18^ Department of Dermatology, University Hospital Tübingen, Tübingen, Germany

**Keywords:** brain metastases, BRAF, immune checkpoint inhibitor (ICI), targeted therapy, melanoma, long-term survival

## Abstract

**Background:**

Modern therapeutic strategies have significantly improved the prognosis of advanced melanoma patients. Predictive factors of therapy response include serum LDH; however, predictive markers for long-term survival are currently largely lacking.

**Patients and methods:**

Patients diagnosed with stage IV melanoma (AJCCv8) of cutaneous origin or unknown primary were identified from the prospective multicenter German Dermatologic Cooperative Oncology Group (DeCOG) skin cancer registry ADOREG. Baseline characteristics were compared between patient groups with short-term versus long-term survival. Statistical analysis included ROC analysis and multinomial regression analysis.

**Results:**

Of 3066 stage IV melanoma patients entered into the ADOREG between 05/2014 and 06/2021, 395 were identified for this study, of whom 301 (76.2%) survived ≤1 year, and 94 (23.8%) survived ≥5 years after stage IV diagnosis. The median follow-up time was 6 months (range 0-129 months). Regarding the baseline characteristics, only elevated serum LDH (P <0.001) was found to be independently predicting survival ≤1 year. Type of first-line therapy, immune checkpoint inhibition (ICI) versus BRAF/MEK targeted therapy (TT), was not predictive of long-term survival ≥5 years. For survival ≤1 year, the presence of brain metastases at treatment start was an independent predictor in BRAF-mutated patients regardless if they received TT (N=113; P=0<0.001) or ICI (N=69; P=0.015), but not in BRAF-wildtype patients who received ICI (N=161; P=0.47).

**Conclusions:**

Low serum LDH independently predicts long-term survival of stage IV melanoma patients in every subgroup of treatment type and BRAF status. Brain metastasis has a negative impact on long-term survival in BRAF-mutated, but not in BRAF-wildtype patients. Investigation of molecular features of brain metastases in BRAF-mutated vs. BRAF-wildtype melanomas may lead to new insights in tumor biology and may yield new therapeutic approaches.

## Introduction

Melanoma is one of the deadliest skin cancer types (1). With the introduction of modern therapy strategies such as immune checkpoint inhibition (ICI) and BRAF/MEK-directed targeted therapy (TT), the prognosis of patients with advanced melanoma has improved significantly with response rates up to 70% and 5-year overall survival rates up to 50% (2–5). After the introduction of these modern systemic therapies, the initial focus of research was on factors that determine a good or poor treatment outcome.

For both, ICI and TT, there is now an increasing amount of long-term survival data available from randomized clinical trials (RCT). Analysis of these data demonstrate that patient survival curves plateau after only 3-5 years after initiation of therapy (6, 7). Therefore, there is an increasing interest in identifying the factors that are determinants of a patient’s long-term survival or even cure (8). Especially from RCT study results, we know that at treatment baseline, an overall low tumor burden with low serum lactate dehydrogenase (LDH), as well as the absence of brain metastasis correlates positively with long-term patient survival upon the respective first-line therapy (9). In contrast, studies on real-world data on factors affecting long-term survival of melanoma patients with distant metastases are rare.

The aim of the present study was to identify factors predictive for long-term versus short-term survival of a real-world cohort of stage IV melanoma patients after onset of a modern first-line therapy with PD-1-based ICI or BRAF/MEK-directed TT.

## Patients and methods

### Study design

Patients with histologically confirmed melanoma of the skin or of unknown primary (MUP) diagnosed with stage IV by AJCCv8 and started a first-line systemic treatment between May 2010 and October 2021. From the prospective multicenter skin cancer registry ADOREG of the German Dermatological Cooperative Oncology Group (DeCOG), only patients who were alive either ≤1 year or ≥5 years after stage IV diagnosis were included. The study endpoint was overall survival (OS), defined as time after start of first-line therapy in stage IV and death of any cause. Patient and tumor characteristics at baseline of first-line therapy in stage IV, sex (m vs f), age (≤ vs > 65 years), M stage by AJCCv8 (M1a/b vs M1c/d), serum LDH, numbers of organs involved with metastasis (<3 vs ≥3), and presence of specific organ metastasis (lung, liver and brain) were analyzed for distribution and association to OS. This analysis was performed on the total patient cohort, as well as on patient subgroups subdivided by type of first-line therapy (PD-1-based ICI, and BRAF/MEK-directed TT) and tumor BRAF mutation status. All patients gave written informed consent before documentation of their data in the ADOREG registry. The ADOREG registry was approved by the ethics committee of the University Duisburg-Essen (15-6566-BO).

### Statistical analysis

The chi-square test, student’s t-test, receiver operating curves (ROC) analysis, and multinomial logistic regression analysis were performed to investigate the effects of baseline patient and tumor characteristics, as well as therapy selection, on patient survival (OS). P<0.05 was considered statistically significant. Univariate statistical analysis consisting of chi-square test, student’s t-test, ROC analysis, and multivariate analysis were performed with SPSS (Version 25, IBM, Armonk, NY, USA).

## Results

### Patient characteristics

**Figure 1:**
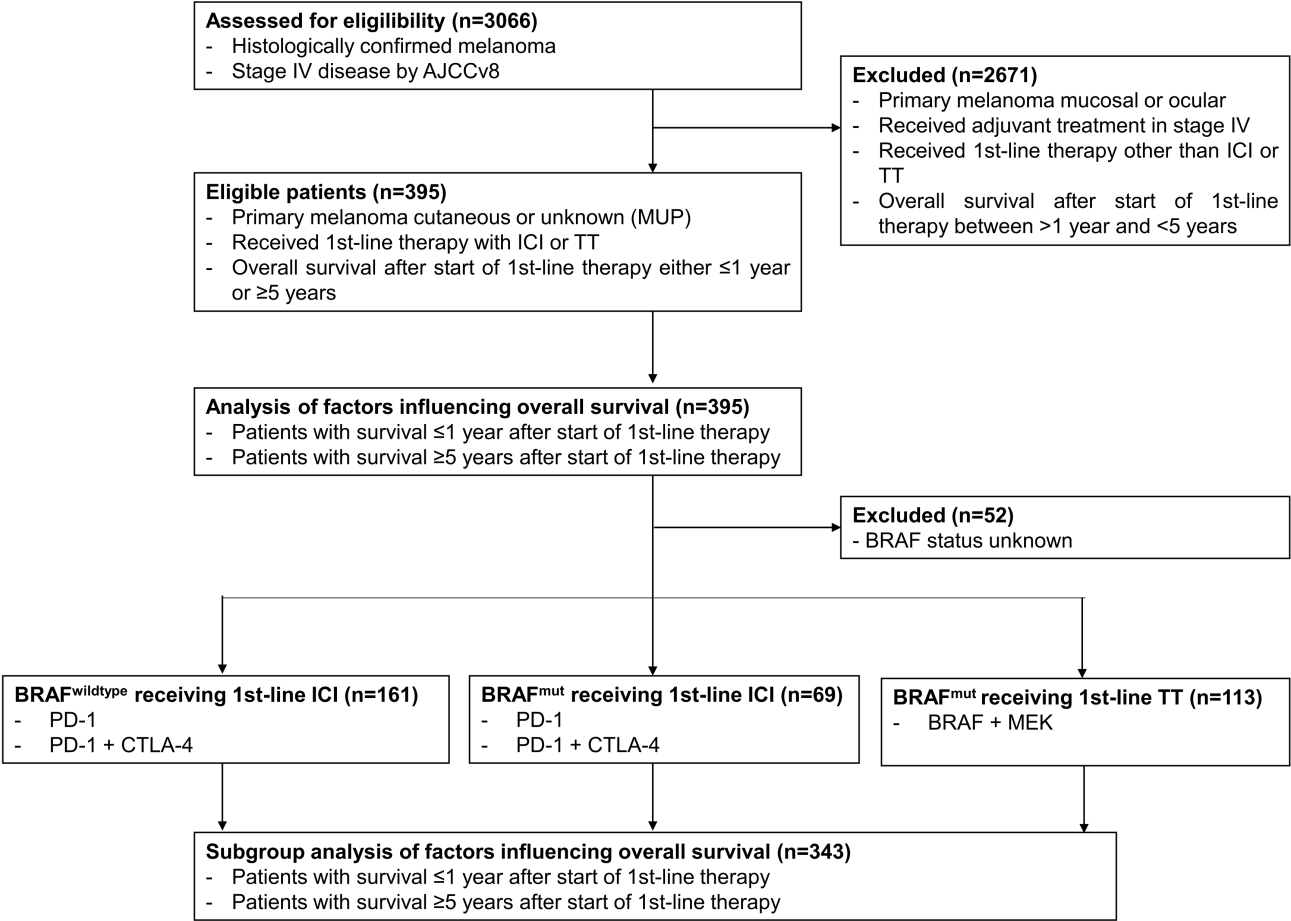
Schematic presentation of the study flow.

Data cut-off was February 2022. Of the patients enrolled into the ADOREG at that time, 3066 were diagnosed with stage IV melanoma. Of these, 395 patients met the selection criteria for the present study (**Figure 1**). Among them, 237 (60.0%) were men and 158 (40.0%) were women. The mean age of the patients was 64.3 years (range 19-96). 128 patients (32.4%) received BRAF/MEK-directed TT, 174 (44.1%) received PD-1 ICI monotherapy, and 93 (23.5%) received PD-1 plus CTLA-4 dual ICI therapy. Detailed baseline characteristics of the patients and their tumors including tumor subtype, BRAF mutation status, primary tumor thickness (Breslow), and primary tumor ulceration are listed in **Table 1**.

**Table 1:** Patient characteristics.

	All patients N (%)	OS after 1L therapy ≤1 year N (%)	OS after 1L therapy ≥5years N (%)
Total	395 (100%)	301 (100%)	94 (100%)
Sex			
Male	237 (60.0%)	182 (60.5%)	55 (58.5%)
Female	158 (40.0%)	119 (39.5%)	39 (41.5%)
Mean age, years (range)	64,3 (19-96)	65.0 (19-96)	62.2 (29-84)
Tumor subtype			
ALM	22 (5.7%)	21 (7.0%)	1 (1.1%)
LLM	9 (2.7%)	9 (3.0%)	0 (0%)
UCM	85 (21.5%)	65 (21.7%)	20 (21.3%)
NM	136 (34.4%)	104 (34.0%)	32 (34.0%)
SSM	87 (22.0%)	65 (21.7%)	22 (23.4%)
MUP	56 (14.2%)	37(12.4%)	19 (20.2%)
ECOG			
0	137 (34.7%)	105 (31.1%)	39 (41.5%)
≥1	121 (30.6%)	107 (31.7%)	19 (20.2%)
Unknown	137 (34,7%)	126 (37.3%)	36 (38.3%)
Mean tumor thickness, mm (range)	4.9 (0 - 55)	5.2 (0-55)	3.9 (0 - 14)
Ulceration of primary			
Yes	166 (42.0%)	134 (44.5%)	32 (34.0%)
No	130 (32.9%)	96 (31.9%)	34 (36.2%)
Unknown	99 (25.1%)	71 (23.5%)	28 (29.8%)
BRAF status			
Mutation	182 (46.1%)	125 (41.5%)	57 (60.6%)
No Mutation	161 (40.7%)	130 (43.2%)	31 (33.0%)
Unknown	52 (13.2%)	46 (15.3%)	6 (6.4%)
Serum LDH No. (%)			
Normal	130 (32.9%)	76 (25.2%)	54 (57.4%)
Elevated	195 (49.4%)	171 (56.8%)	24 (25.5%)
Unknown	70 (17.7%)	54 (17.9%)	16 (17.1%)
M Stage			
M1a or M1b	70 (17.7%)	39 (13%)	31 (33%)
M1c or M1d	315 (79.7%)	253 (84.1%)	62 (66%)
Unknown	10 (2.5%)	9 (3%)	1 (1.1%)
First-line therapy			
BRAF/MEK targeted therapy	128 (32.4%)	92 (30.6%)	36 (38.3%)
PD-1 mono ICI	174 (44.1%)	125 (41.5%)	49 (52.1%)
PD-1 + CTLA-4 dual ICI	93 (23.5%)	84 (27.9%)	9 (9.6%)

Characteristics of melanoma patients at first diagnosis of stage IV disease, prior to the start of first-line treatment. Disease staging was performed according to AJCCv8.

### Patients with elevated serum LDH are less likely to show long-term survival

**Figure 2:**
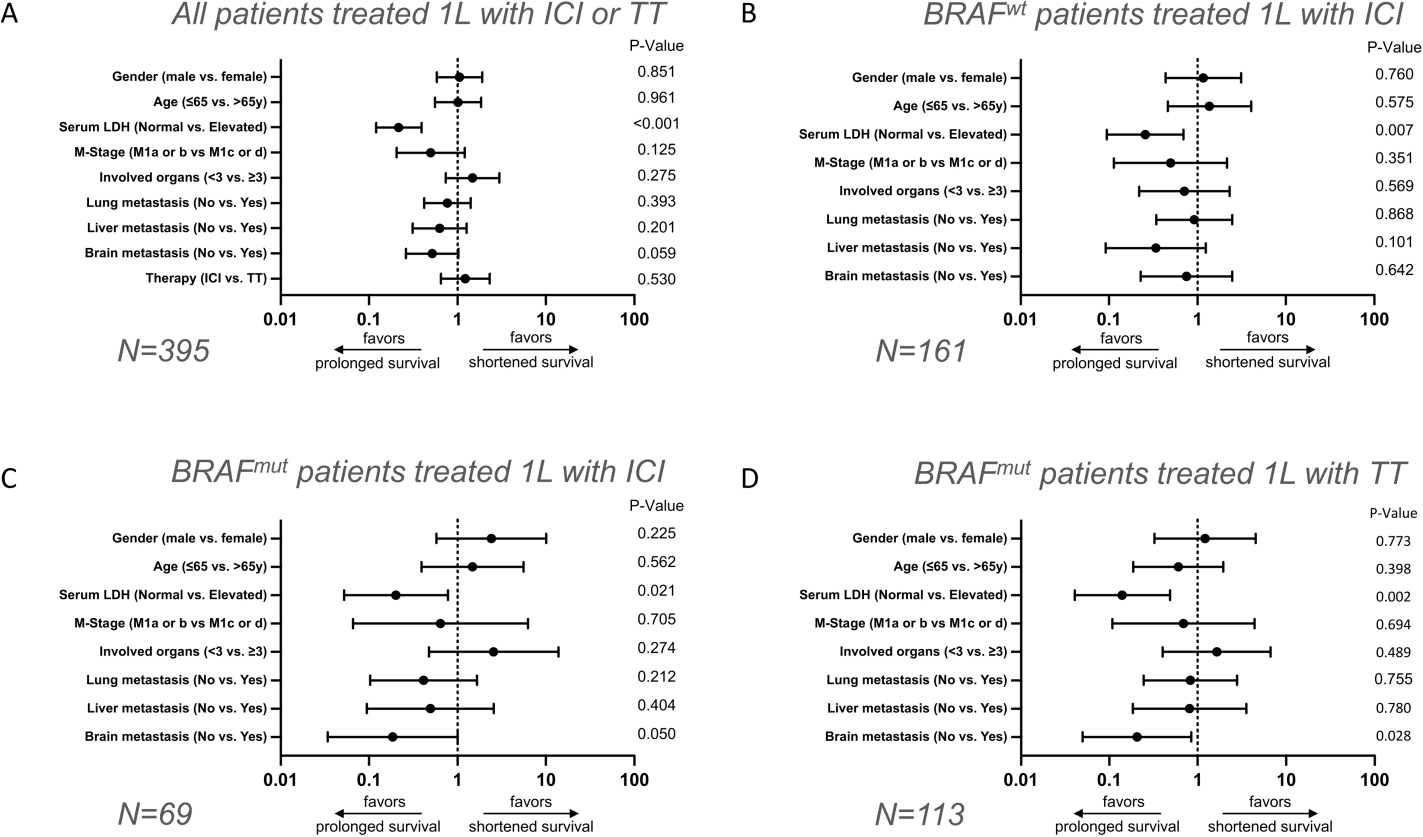
Multivariate analysis of baseline clinical parameters on long-term versus short-term survival after 1L therapy in stage IV melanoma patients. Forest plots show multivariate evaluation of clinical parameters associated with long-term OS ≥5 years versus short-term OS ≤1 year in melanoma patients who received ICI or TT **(A)**, BRAF-wildtype patients who received ICI **(B)**, BRAF-mutant patients who received ICI **(C)**, and BRAF-mutant patients who received TT **(D)**.

In order to determine which baseline factors affect long-term survival in stage IV melanoma patients we first performed multinomial regressions. Due to the high number of missing values, ECOG, tumor thickness and tumor ulceration status were excluded from the analysis. First, the entire patient cohort was analyzed regardless of the type of therapy received. Among the included factors, a statistically significant negative association was only found between a high serum LDH level (HR=4.619 CI=2.550 - 8.368; P<0.001) and long-term survival ≥5 years. The other included factors such as therapy (ICI vs. TT) (HR=0.816 CI=0.433 – 1.539; P=0.530), brain metastases (no vs. yes) (HR=1.928 CI=0.975 – 3.184; P=0.059), liver metastases (no vs. yes) (HR=1.587 CI=0.782 – 3.222; P=0.201), lung metastases (no vs. yes) (HR=1.302 CI=0.711 – 2.386; P=0.393), number of organs involved (<3 vs. ≥3) (HR=0.337 CI=0.337 – 1.367; P=0.275), M category (M1a or b vs. c or d) (HR=2.012 CI=0.824 – 4.913; P=0.125), age (<65 vs. ≥65) (HR=0.985 CI=0.541 – 1.794; P=0.961) and gender (male vs. female) (HR=0.948 CI=0.523 – 1.717; P=0.859) showed no statistically significant effect on long-term survival (OS ≥5 years) (**Figure 2A**, **Supplementary Table 1**).

To investigate the extent to which factors influence long-term survival in relation to the type of first-line therapy and BRAF status, the total patient cohort was subdivided into three subgroups. The first subgroup included BRAF-wildtype patients who received first-line ICI. Again, only elevated serum LDH (HR=3.887; CI=1.440–10.490; P=0.007) had a statistically significant negative impact on long-term survival (OS ≥5 years). All other factors investigated, such as brain metastases (no vs. yes; HR=1.326; CI=0.403–4.360; P=0.642), liver metastases (no vs. yes; HR=2.959; CI=0.808–10.841; P=0.101), lung metastases (no vs. yes; HR=1.088; CI=0.403–2.936; P=0.868), number of organs involved with metastasis (<3 vs. ≥3; HR=1.409; CI=0.434–4.575; P=0.569), M category (M1a/b vs. c/d; HR=2.014; CI=0.462–8.776; P=0.351), age (<65 vs. ≥65 years; HR=0.733; CI=0.247-2.173; P=0.575) and sex (male vs. female; HR=0.858; CI=0.322–2.288; P=0.760) indicated no statistically significant effect on the relative chance of long-term survival (**Figure 2B**, **Supplementary Table 2**).

**Figure 3:**
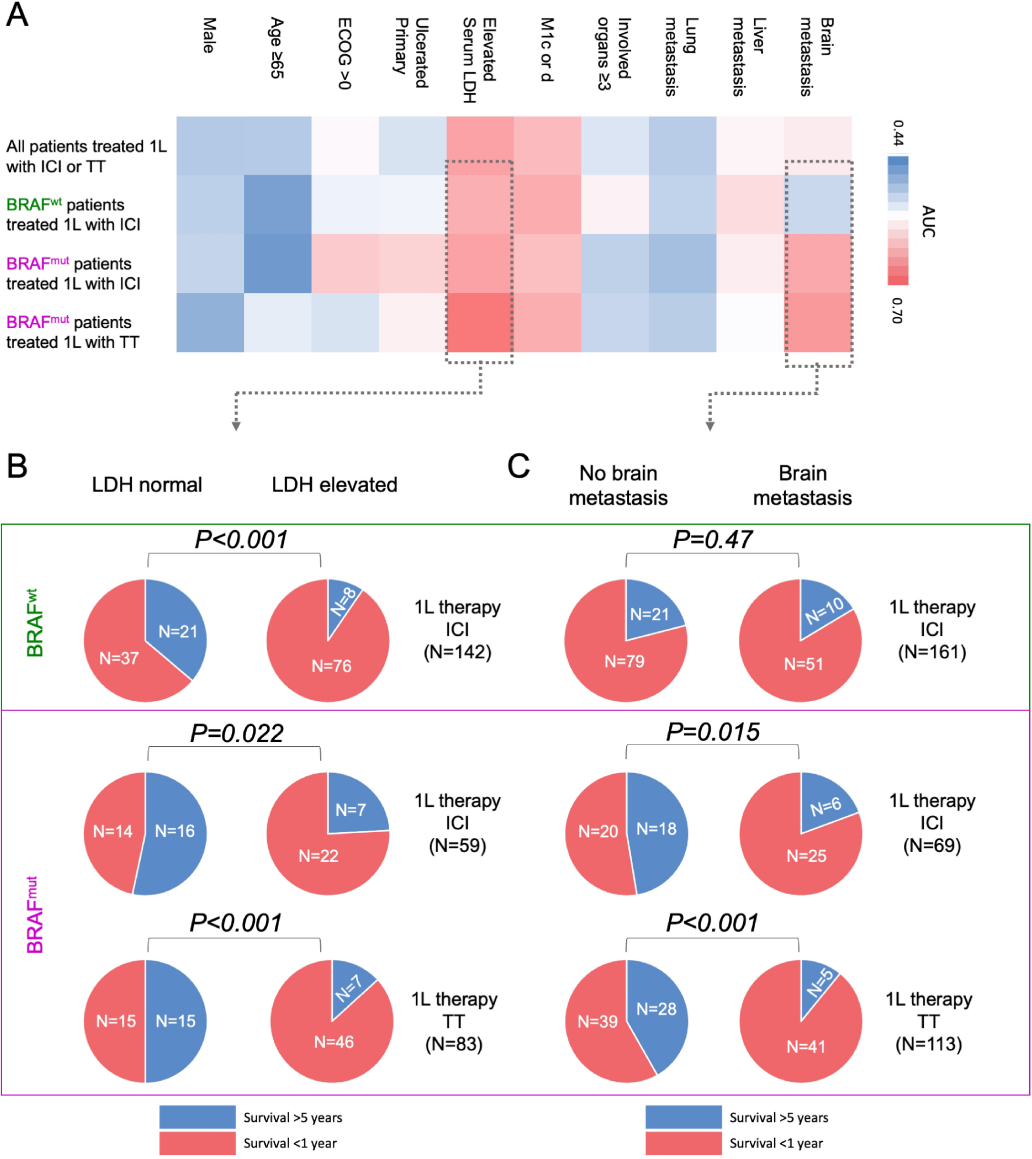
Impact of clinical parameters on long-term versus short-term survival after 1L therapy in stage IV melanoma patients by univariate analysis. **(A)** The heatmap shows the univariate evaluation of clinical parameters associated with long-term OS ≥5 years versus short-term OS ≤1 year depending on therapy type and BRAF status. Pie charts show the impact of **(B)** serum LDH, and **(C)** presence of brain metastasis on long-term OS ≥5 years versus short-term OS ≤1 year in BRAF-wildtype patients (top, green) who received ICI versus BRAF-mutant patients who received ICI or TT (bottom, purple).

In contrast, in the subgroup of BRAF-mutant patients receiving ICI a negative impact of the presence of brain metastasis (HR=5.391; CI=0.998–29.118; P=0.05) was found on long-term survival (OS ≥5 years), in addition to a negative impact of elevated serum LDH (HR=4.973; CI=1.279–19.341; P=0.021); **Figures 2C**, **3**. The other factors included such as liver metastases (no vs. yes; HR=2.021; CI=0.387–10.541; P=0.404), lung metastases (no vs. yes; HR=2.422; CI=0.603–9.721; P=0.212), number of organs affected (<3 vs. ≥3; HR=0.390; CI=0.072–2.103; P=0.274), M category (M1a/B vs. c/d; HR=1.533; CI=0.159–15.140; P=0.705), age (<65 vs. ≥65; HR=0.676; CI=0.180-2.542; P=0.562), sex (male vs. female; HR=0.413; CI=0.099–1.723; P=0.225) showed no statistically significant effect on long-term survival (**Figure 2C**, **Supplementary Table 3**).

Last, the group of BRAF-mutant patients under TT was considered. Here again, an elevated serum LDH level was associated with poor long-term survival (HR=7.124; CI=2.066–24.572; P=0.002). Similarly, the presence of brain metastases was associated with poor OS (HR=4.854; CI=1.186–19.869; P=0.028). The other factors included such as liver metastases (no vs. yes; HR=1.235; CI=0.281–5.439; P=0.780), lung metastases (no vs. yes; HR=1.214; CI=0.360–4.095; P=0.755), number of organs affected (<3 vs. ≥3; HR=0.609; CI=0.149–2.484; P=0.489), M category (M1a/b vs. c/d; HR=1.447; CI=0.229–9.157; P=0.694), age (<65 vs. ≥65 years; HR=1.659; CI=0.513–5.372; P=0.398), sex (male vs. female; HR=0.824; CI=0.220-3.078; P=0.773) had no statistically significant effect on the relative probability of long-term survival (**Figure 2D**, **Supplementary Table 4**).

### Presence of brain metastases is differentially associated with long-term survival dependent on BRAF mutation status

Multiple receiver operating characteristic (ROC) curve analyses were conducted on the four distinct patient subgroups to corroborate the findings derived from the multivariate analysis. Compared with the other factors tested, increased serum LDH was again negatively associated with long-term survival (OS ≥5 years) in all subgroups; **Figure 3A**, **Supplementary Table 5**). Similary to the results from the multinomial regression, ROC analyses found a different survival impact of the presence of brain metastases between BRAF-mutant and BRAF-wildtype melanoma patients. In BRAF-wildtype patients who received ICI, ROC analysis showed no effect of brain metastasis on long-term survival (AUC=0.523, P=0.624). In contrast, for BRAF-mutant patients a negative association between the presence of brain metastasis and long-term survival could be detected, regardless of whether patients received ICI (AUC=0.640; P=0.047) or TT (AUC=0.655; P=0.005) (**Figure 3A**, **Supplementary Table 5**).

In a next step, the absolute distribution of patients with normal vs. elevated serum LDH, and absent vs. present brain metastases was considered in terms of long-term vs short-term OS after 1st-line therapy in the total patient cohort as well as in the subgroups. Looking at the distribution of normal versus elevated serum LDH, we found that in BRAF-wildtype patients treated with ICI, patients with normal serum LDH were significantly more likely to survive ≥5 years (P<0.001; **Figure 3B**). This association could be detected in a similar extent in BRAF-mutant patients, independent of their type of treatment. Thus, BRAF-mutant patients with normal serum LDH at baseline were significantly more likely to survive ≥5 years when treated with ICI (P=0.022). BRAF-mutant patients with normal serum LDH treated with TT also showed significantly higher probability to survive ≥5 years (P<0.001; **Figure 3B**). In contrast, associations were differentially distributed when considering the presence or absence of brain metastasis. In BRAF-wildtype patients treated with ICI, there was no statistically significant difference between patients with or without brain metastases in terms of OS ≥5 years after therapy start (P=0.472; **Figure 3B**). However, in BRAF-mutant patients, the presence of brain metastasis had a significant impact on long-term survival independent of treatment type. Thus, in BRAF-mutant patients treated with ICI, patients without brain metastases were significantly more likely to survive ≥5 years (P=0.015). Similarly, BRAF-mutant patients treated with TT had a significantly higher probability of survival ≥5 years if they started treatment without brain metastases (P<0.001; **Figure 3C**).

## Discussion

The introduction of modern systemic therapies such as BRAF/MEK-directed TT and PD-1-based ICI has led to a significant improvement in overall survival of stage IV melanoma patients with distant metastases (6, 10). In the past, numerous influencing clinical baseline parameters such as ECOG, elevated serum LDH, brain and liver metastases, among others, have been identified to be associated with poorer treatment response, and a shortened progression-free and overall survival (11–14). The data on long-term overall survival of stage IV melanoma patients is currently limited, with published data mostly restricted to analyses of data from clinical trials (RCT). For example, a study published in 2019 examined the long-term survival of BRAF-mutant patients treated with the BRAF/MEK inhibitors dabrafenib and trametinib, and identified performance status, age, sex, number of organ sites with metastasis, and LDH serum level as predictors of survival ≥5 years (6).

In the present study, we investigated real-world patient data from stage IV melanoma patients for predictors of particular survival groups, long-term (≥5 years) versus short-term (≤1 year) OS, and examined differences in special patient subgroups by type of first-line therapy and tumor BRAF mutation status. Regardless of the presence of a BRAF mutation, an elevated serum LDH at baseline was the most significant predictor for a particularly early death, defined as an OS less than one year. An increase in serum LDH level indicates higher metabolic activity of tumor cells and correlates with a higher tumor burden. It is well established that elevated serum LDH levels are associated with poorer patient outcomes, as demonstrated in almost every registration study (4, 6, 10–12). Further subdividing the investigated patients into subgroups by type of first-line therapy, the presence of brain metastases was an independent predictor for survival ≤1 year in BRAF-mutated patients regardless if they received BRAF/MEK-directed TT or PD-1-based ICI. Surprisingly, this association could not be detected in BRAF-wildtype melanoma patients who received ICI.

Interestingly, we found that the presence of a BRAF mutation significantly impacts the long-term survival of melanoma patients with brain metastasis. Specifically, a notable decrease in the probability of achieving long-term survival of more than 5 years was observed in patients with brain metastases harboring a BRAF mutation compared to their wildtype counterparts. One explanation could be a fundamentally higher aggressiveness of BRAF-mutant melanomas, which has been described in several studies conducted prior to the introduction of targeted tumor therapy (15, 16). The data of this investigation correlate with a study from 2023 in which the authors observed that patients with BRAF–mutated melanoma had a low mutational burden in tumor tissue from brain metastases. On a molecular level the study showed a lower infiltration of immune cells in brain metastases of BRAF-mutant patients compared to BRAF-wildtype patients and a resulting significantly shorter survival time in BRAF-mutant patients with brain metastases compared to BRAF-wildtype patients with brain metastases (17).

Our present study and its statistical analysis have some limitations. First, the study is based on real-world patient data, which means that the compared treatment groups are not stratified or balanced. Second, both statistical analysis procedures using chi-square tests and ROC analyses are univariate analyses. With these, there is a risk of bias due to other clinical parameters. Therefore, an additional multivariate analysis was performed. Another limitation is the partial lack of data for some clinical parameters such as ECOG and histopathology. A further limitation of this study is the inclusion of patients treated between May 2010 and October 2021, with a data cutoff in February 2022, which prevents a true evaluation of five-year survival for the most recently treated patients. This is of particular interest, as the approved treatment modalities have changed dramatically over this period.

Taken together, our study results show that serum LDH predicts long-term survival of stage IV melanoma patients independently of treatment type and BRAF mutation status. Brain metastasis has a relevant impact on long-term survival in BRAF-mutated, but not in BRAF-wildtype patients. Investigations correlating additional clinical factors, such as the location, size, and number of brain metastases, with the intracranial therapeutic response in BRAF-mutated and BRAF wild-type patients are urgently needed. Investigation of molecular features of brain metastases in BRAF-mutated vs. BRAF-wildtype melanomas may lead to new insights in tumor biology and may yield new therapeutic approaches. In the future, there may be potential to identify new drug targets for therapy or prevention of brain metastases in melanoma patients by conducting protein expression studies using transcriptomic or proteomic approaches.

## Data Availability

The raw data supporting the conclusions of this article will be made available by the authors, without undue reservation.

## Supplementary material

Supplementary Table 1 Multivariate analysis on baseline characteristics with regard to longterm overall survival. Multinomial regression analysis examining the impact of baseline characteristics on OS ≥5 years after start of 1L ICI or TT therapy in n=280 stage IV melanoma patients.

Supplementary Table 2 Multivariate analysis on baseline characteristics with regard to longterm overall survival (BRAF wildtype). Multinomial regression analysis examining the impact of baseline characteristics on OS ≥5 years after start of 1L ICI therapy in n=139 stage IV BRAF-wildtype melanoma patients.

Supplementary Table 3 Multivariate analysis on baseline characteristics with regard to longterm overall survival (BRAF mutant). Multinomial regression analysis examining the impact of baseline characteristics on OS ≥5 years after start of 1L ICI therapy in n=59 stage IV BRAF-mutnat melanoma patients.

Supplementary Table 4 Multinomial regression analysis examining the impact of baseline characteristics on OS ≥5 years after start of 1L TT therapy in n=82 stage IV BRAF-mutant melanoma patients.

Supplementary Table 5 ROC analysis for OS ≥5 years in total patients and subgroups.
