# Supplementary material for: Presence of brain metastasis differentially impacts long-term survival after first-line therapy in melanoma depending on BRAF mutation status
Source: Front Immunol. 2025 Feb 14;16:1536642. doi: 10.3389/fimmu.2025.1536642 (PMC11868123; doi:10.3389/fimmu.2025.1536642)
Supplement: Supplementary Table 1 — Multivariate analysis on baseline characteristics with regard to longterm overall survival. Multinomial regression analysis examining the impact of baseline characteristics on OS ≥5 years after start of 1L ICI or TT therapy in n=280 stage IV melanoma patients. [file DataSheet1.docx]

Supplementary Table 1. Multivariate analysis on baseline characteristics with regard to longterm overall survival.

|  | **OS after 1L therapy ≥5 years** | |
| --- | --- | --- |
| **Parameters included** | **Hazard ratio (95% CI)** | ***p*-value** |
| **Sex** | 0.948 (0.523 – 1.717) | 0.859 |
| (male *versus* female) |  |  |
| **Age** | 0.985 (0.541 – 1.794) | 0.961 |
| (<65 *versus* ≥65) |  |  |
| **Serum LDH** | 4.619 (2.550 – 8.368) | **<0.001** |
| (normal *versus* elevated) |  |  |
| **M stage** | 2.012 (0.824 – 4.913) | 0.125 |
| (M1a/b *versus* M1c/d) |  |  |
| **Number of organs involved with metastasis**  (<3 *versus* ≥ 3) | 0.678 (0.337 – 1.367) | 0.275 |
|  |  |  |
| **Lung metastasis** | 1.302 (0.711– 2.386) | 0.393 |
| (yes *versus* no) |  |  |
| **Liver metastasis**  (yes *versus* no) | 1.587 (0.782 – 3.222) | 0.201 |
| **Brain metastasis**  (yes *versus* no) | 1.928 (0.975 – 3.184) | 0.059 |
| **Therapy**  (ICI *versus* TT) | 0.816 (0.433– 1.539) | 0.530 |

Multinomial regression analysis examining the impact of baseline characteristics on OS ≥5 years after start of 1L ICI or TT therapy in n=280 stage IV melanoma patients.

**Supplementary Table 2. Multivariate analysis on baseline characteristics with regard to longterm overall survival (BRAF wildtype).**

|  | **OS after 1L therapy ≥5 years** | |
| --- | --- | --- |
| **Parameters included** | **Hazard ratio (95% CI)** | ***p*-value** |
| **Sex** | 0.858 (0.322 – 2.288) | 0.760 |
| (male *versus* female) |  |  |
| **Age** | 0.733 (0.247 – 2.173) | 0.575 |
| (<65 *versus* ≥65) |  |  |
| **Serum LDH** | 3.887 (1.440 – 10.490) | **0.007** |
| (normal *versus* elevated) |  |  |
| **M stage** | 2.014 (0.462 – 8.776) | 0.351 |
| (M1a/b *versus* M1c/d) |  |  |
| **Number of organs involved with metastasis**  (<3 *versus* ≥ 3) | 1.409 (0.434 – 4.575) | 0.569 |
|  |  |  |
| **Lung metastasis** | 1.088 (0.403 – 2.936) | 0.868 |
| (yes *versus* no) |  |  |
| **Liver metastasis**  (yes *versus* no) | 2.959 (0.808 – 10.841) | 0.101 |
| **Brain metastasis**  (yes *versus* no) | 1.326 (0.403 – 4.360) | 0.642 |

Multinomial regression analysis examining the impact of baseline characteristics on OS ≥5 years after start of 1L ICI therapy in n=139 stage IV BRAF-wildtype melanoma patients.

**Supplementary Table 3. Multivariate analysis on baseline characteristics with regard to longterm overall survival (BRAF mutant).**

|  | **OS after 1L therapy ≥5 years** | |
| --- | --- | --- |
| **Parameters included** | **Hazard ratio (95% CI)** | ***p*-value** |
| **Sex** | 0.413 (0.099 – 1.723) | 0.225 |
| (male *versus* female) |  |  |
| **Age** | 0.676 (0.180 – 2.542) | 0.562 |
| (<65 *versus* ≥65) |  |  |
| **Serum LDH** | 4.973 (1.279 – 19.341) | **0.021** |
| (normal *versus* elevated) |  |  |
| **M stage** | 1.553 (0.159 – 15.140) | 0.705 |
| (M1a/b *versus* M1c/d) |  |  |
| **Number of organs involved with metastasis**  (<3 *versus* ≥ 3) | 0.390 (0.072 – 2.103) | 0.274 |
|  |  |  |
| **Lung metastasis** | 2.422 (0.603 – 9.721) | 0.212 |
| (yes *versus* no) |  |  |
| **Liver metastasis**  (yes *versus* no) | 2.021 (0.387 – 10.541) | 0.404 |
| **Brain metastasis**  (yes *versus* no) | 5.391 (0.998 – 29.118) | **0.050** |

Multinomial regression analysis examining the impact of baseline characteristics on OS ≥5 years after start of 1L ICI therapy in n=59 stage IV BRAF-mutnat melanoma patients.

**Supplementary Table 4**

|  | **Longterm survival >5 years** | |
| --- | --- | --- |
| **Parameters included** | **Hazard ratio (95% CI)** | ***p*-value** |
| **Sex** | 0.824 (0.220 – 3.078) | 0.773 |
| (male *versus* female) |  |  |
| **Age** | 1.659 (0.513 – 5.372) | 0.398 |
| (<65 *versus* ≥65) |  |  |
| **Serum LDH** | 7.124 (2.066 – 24.572) | **0.002** |
| (normal *versus* elevated) |  |  |
| **M stage** | 1.447 (0.229 – 9.157) | 0.694 |
| (M1a/b *versus* M1c/d) |  |  |
| **Number of organs involved with metastasis**  (<3 *versus* ≥ 3) | 0.609 (0.149 – 2.484) | 0.489 |
|  |  |  |
|  |  |  |
| **Lung metastasis** | 1.214 (0.360 – 4.095) | 0.755 |
| (yes *versus* no) |  |  |
| **Liver metastasis**  (yes *versus* no) | 1.235 (0.281 – 5.439) | 0.780 |
| **Brain metastasis** | 4.854 (1.186 – 19.869) | **0.028** |
| (yes *versus* no) |  |  |

Multinomial regression analysis examining the impact of baseline characteristics on OS ≥5 years after start of 1L TT therapy in n=82 stage IV BRAF-mutant melanoma patients.

**Supplementary Table 5. ROC analysis for OS ≥5 years in total patients and subgroups.**

|  | **Sex**  **(Male)** | **Age**  **(≥65 years)** | **ECOG**  **(>0)** | **Primary ulceration**  **(yes)** | **Serum LDH**  **(elevated)** | **M stage (M1c/d)** | **Involved organs (≥3)** | **Lung metastasis**  **(yes)** | **Liver metastasis**  **(yes)** | **Brain metastasis**  **(yes)** |
| --- | --- | --- | --- | --- | --- | --- | --- | --- | --- | --- |
| Patients treated with ICI or TT | AUC=0.507  P=0.804 | AUC=0.508 P=0.780 | AUC=0.564 P=0.077 | AUC=0.534 P=0.310 | AUC=0.646 **P<0.001** | AUC=0.623 **P=0.001** | AUC=0.536 P=0.228 | AUC=0.510 P=0.742 | AUC=0.568 **P=0.022** | AUC=0.577 **P=0.009** |
| Patients treated with ICI BRAF WT | AUC=0.514  P=0.766 | AUC=0.464 P=0.457 | AUC=0.550 P=0.354 | AUC=0.552 P=0.354 | AUC=0.633 **P=0.007** | AUC=0.637 **P=0.021** | AUC=0.570 P=0.130 | AUC=0.517 P=0.721 | AUC=0.591 P=0.051 | AUC=0.523 P=0.624 |
| Patients treated with ICI BRAF mutant | AUC=0.519  P=0.796 | AUC=0.457 P=0.540 | AUC=0.608 P=0.198 | AUC=0.600 P=0.242 | AUC=0.646 P=0.054 | AUC=0.619 P=0.163 | AUC=0.515 P=0.829 | AUC=0.496 P=0.957 | AUC=0.575 P=0.307 | AUC=0.640 **P=0.047** |
| Patients treated with TT BRAF mutant | AUC=0.482  P=0.738 | AUC=0.545 P=0.422 | AUC=0.534 P=0.652 | AUC=0.572 P=0.267 | AUC=0.686 **P=0.005** | AUC=0.635 P=0.094 | AUC=0.521 P=0.711 | AUC=0.511 P=0.834 | AUC=0.560 P=0.279 | AUC=0.655 **P=0.005** |
